# A dataset on potentially groundwater-dependent vegetation in the Sierra Nevada Protected Area (Southern Spain) and its underlying NDVI-derived ecohydrological attributes

**DOI:** 10.1016/j.dib.2025.111760

**Published:** 2025-06-09

**Authors:** Javier Cabello, Montserrat Escudero-Clares, Sergio Martos-Rosillo, J. Jesús Casas, Juanma Cintas, Thomas Zakaluk, María J. Salinas-Bonillo

**Affiliations:** a Andalusian Center for Global Change (ENGLOBA), Ctra. de Sacramento s/n, La Cañada de San Urbano, 04120, Almería Spain; b Dept. of Biology and Geology, University of Almería, Ctra. de Sacramento s/n, La Cañada de San Urbano, 04120, Almería Spain; c IGME-CSIC, Geological and Mining Institute of Spain - Spanish National Research Council, Urb. Alcázar del Genil, 4-Edif. Zulema, Bajo and 1°C, Granada 18006, Spain; d EEZA, CSIC. Ctra. de Sacramento s/n, La Cañada de San Urbano, 04120, Almería Spain; e Asociación Solamb. Calle Los Naranjos, Alhama de Almería 04400, Spain

**Keywords:** Dryland mountains, Ecohydrology, Groundwater, NDVI time series, Remote sensing

## Abstract

This dataset provides a spatially explicit classification of potentially groundwater-dependent vegetation (pGDV) in the Sierra Nevada Protected Area (Southern Spain), generated using Sentinel-2 imagery (2019–2023) and ecohydrological attributes derived from NDVI time series. NDVI metrics were calculated from cloud- and snow-filtered Sentinel-2 Level 2A images processed in Google Earth Engine. Monthly NDVI values were used to extract three ecohydrological indicators: dry-season NDVI, dry–wet seasonal NDVI difference, and interannual NDVI variability. Based on quartile classifications of these indicators, 64 ecohydrological vegetation classes were defined. These were further clustered into three levels of potential groundwater dependence using hierarchical clustering techniques, differentiating between alpine and lower-elevation aquifer zones.

The dataset includes raster layers (GeoTIFF) of the ecohydrological classes and pGDV types at 10 m spatial resolution, a CSV file with descriptive statistics for each class, and complete metadata. All spatial layers are projected in ETRS89 / UTM Zone 30N (EPSG: 25830) and are ready for visualization and analysis in standard GIS platforms. Partial validation of the classification was performed using spring location data and the distribution of hygrophilous plant species from official conservation databases. This available dataset enables reproducible analysis of vegetation–groundwater relationships in dryland mountain ecosystems. It supports comparative research across regions, facilitates the study of groundwater buffering effects on vegetation function, and offers a transferable framework for ecohydrological classification based on remote sensing. The data can be reused to inform biodiversity conservation, groundwater management, and climate change adaptation strategies in the Mediterranean and other water-limited mountain regions.

Specifications Table

**Table:** 

Subject	Earth & Environmental Sciences
Specific subject area	Ecohydrology*.*
Type of data	GeoTIFF files (raster layers), Shapefiles (vector layers), CSV tables, Metadata documentation. The dataset consists of processed, analyzed, and derived data products
Data collection	Sentinel-2 Level 2A imagery (2019–2023) downloaded from the Copernicus Open Access Hub (https://scihub.copernicus.eu/) was processed in Google Earth Engine to compute NDVI time series, filtering pixels with >10% cloud and >1% snow probability. Areas with NDVI < 0.1 were excluded. Quartiles categorized NDVI-based attributes into 64 ecohydrological classes, then clustered using hierarchical analysis (Euclidean distance, complete linkage) into three pGDV levels. Spatial and statistical analyses were done in QGIS Desktop (v 3-28.4) and R (v4.2.3)*.*
Data source location	Sierra Nevada Protected Area (Natural and National Parks), southern Spain (37.0°–37.2°N, 2.5°–3.5°W) (Fig. 1). Data were generated and processed at the Andalusian Centre for Global Change (Centro Andaluz para el Cambio Global, University of Almería) and are publicly available in Zenodo.
Data accessibility	Repository name: ZENODO Data identification number: 10.5281/zenodo.15315192 Direct URL to data: https://zenodo.org/records/15315192 Instructions for accessing these data: Open access; all files can be freely downloaded from the Zenodo repository.
Related research article	Cabello, J., Escudero-Clares, M., Martos-Rosillo, S., Casas, J. J., Cintas, J., Zakaluk, T., & Salinas-Bonillo, M. J. (2025). Groundwater-dependent vegetation in semi-arid Mediterranean mountains: The hidden role of hard-rock aquifers. Submitted to the Journal of Hydrology*.*

## 1 Value of the Data

• Groundwater-dependent ecosystems (GDVs) provide essential ecosystem services but are increasingly threatened by water overextraction and climate change. Effective conservation of GDVs requires the ability to detect and map vegetation potentially supported by groundwater. Unlike most studies focus solely on vegetation distribution, the dataset enables users to explore how underlying aquifer characteristics (e.g., fractured metamorphic vs. carbonate systems) may shape the persistence and ecological function of GDVs.
• This dataset provides a spatially explicit classification of potentially groundwater-dependent vegetation (pGDV) in a semi-arid Mediterranean mountain system [1]. By linking functional vegetation traits to spatial patterns of groundwater influence, the dataset offers a novel approach to understanding ecohydrological functioning in dryland mountain ecosystems.
• The NDVI-based ecohydrological attributes included in the dataset (dry-season NDVI, dry–wet seasonal NDVI difference, and interannual NDVI variability) allow researchers to assess vegetation resilience and potential groundwater buffering across spatial and temporal scales.
• The dataset supports comparative research across mountain systems by offering a replicable framework to analyze vegetation–groundwater interactions. It facilitates the identification of functional vegetation patterns and their vulnerability to climate-driven changes, contributing to broader assessments of ecosystem resilience in water-limited environments.
• This dataset can inform climate adaptation strategies and conservation by highlighting areas where groundwater maintains ecological stability under increasing drought and snowpack decline in Mediterranean mountain regions.

## 2 Background

Recharge in mountain aquifers through snowmelt and rainfall runoff creates favourable conditions for vegetated areas likely reliant on groundwater [2,3]. However, despite their critical role in the water cycle, research on groundwater-dependent vegetation (GDV) remains limited in mountain environments [4]. This gap is particularly relevant in arid regions, where alpine aquifers [5] and fractured hard-rock aquifers, both with limited storage capacity [6], can sustain mountain vegetation under conditions of low precipitation and high evapotranspiration demand in arid regions [7].

Most studies focus on GDV distribution, overlooking the influence of aquifer types on their persistence and function [e.g., [8], [9], [10]]. Mountain landscapes host diverse aquifer types that differ in recharge mechanisms, retention capacity, and ecological influence. Mapping areas of potential GDV (pGDV) considering aquifer types is essential to understanding spatial patterns of groundwater dependence and assessing GDV vulnerability to climate change. This dataset provides a spatially explicit classification of pGDV in the Sierra Nevada protected area (Southern Spain), based on NDVI-derived ecohydrological indicators and a reclassified vegetation map. The region hosts three hydrological units: Alpine aquifer zone (AAZ), Slope aquifer zone (SAZ), and Carbonate aquifer zone (CAZ). It complements the associated research article by making the data, methods, and classification outputs accessible for reuse, replication, and application in other regions.

## 3 Data Description

**Fig. 1:**
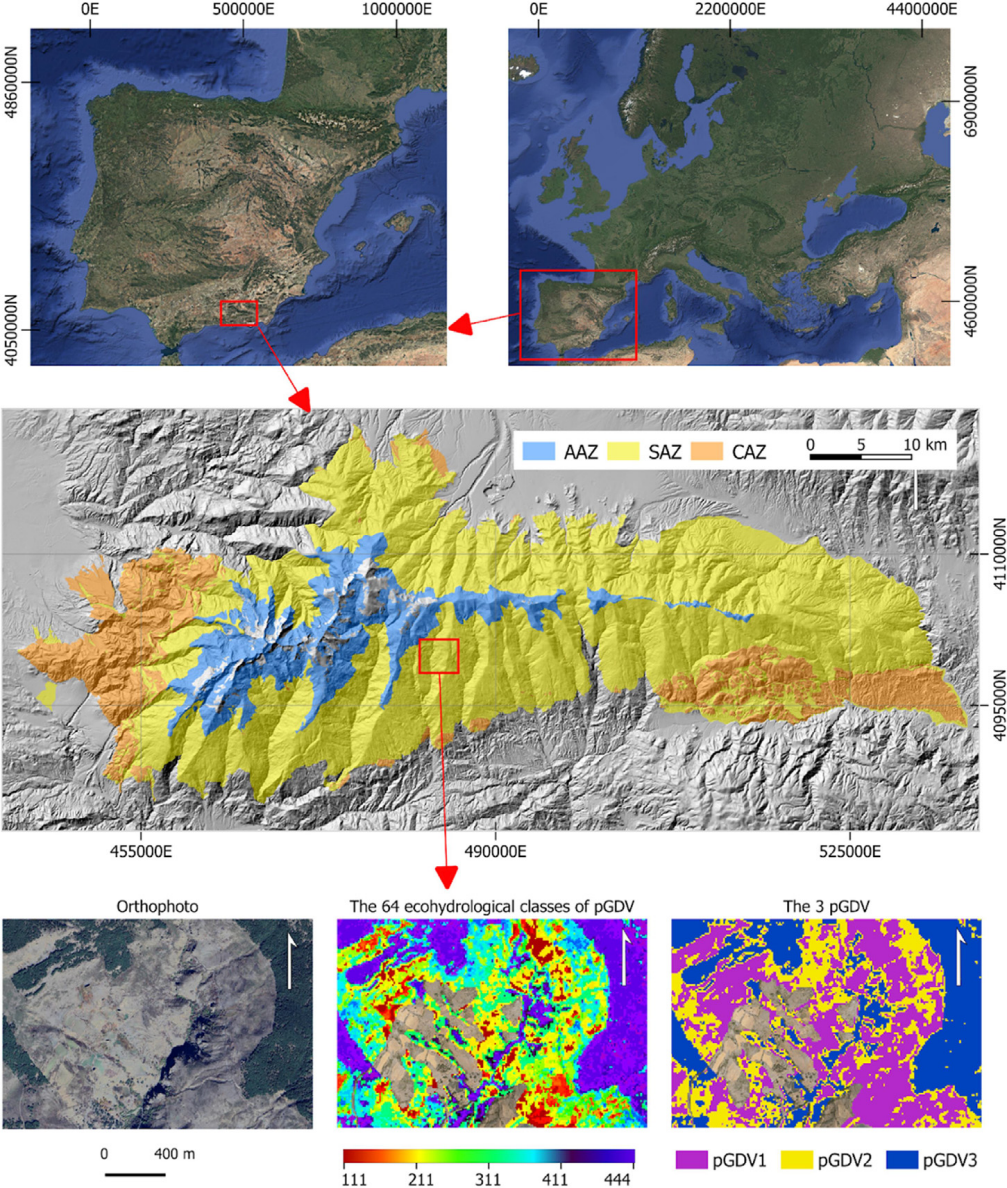
Location and spatial structure of the dataset on groundwater-dependent vegetation (pGDV) in Sierra Nevada, southeastern Spain. Top: location maps at the European and Iberian Peninsula scales. Middle: distribution of aquifers zones (AAZ: alpine–azonal zone, SAZ: subalpine–azonal zone, CAZ: colline–azonal zone) across Sierra Nevada. Bottom: detailed view of a representative area showing (i) orthophoto, (ii) the 64 ecohydrological vegetation classes (pGDV) derived from structural and functional (NDVI-based) characteristics, and (iii) a simplified classification into three main pGDV types.

The dataset is organized into several files that provide the classification of potentially groundwater-dependent vegetation (pGDV) in the Sierra Nevada Protected Area and the ecohydrological indicators used to generate it [11] (Fig. 1). The files are provided in GeoTIFF and CSV formats, accompanied by metadata in PDF and TXT format. The dataset includes:

• pGDV_map_SierraNevada_10m.tif: raster layer (GeoTIFF) showing the final classification of vegetation into three levels of potential groundwater dependence (pGDV1: low, pGDV2: moderate, pGDV3: high), at 10 m spatial resolution.
• ecoHydro64_classes_SierraNevada_10m.tif: raster layer with 64 ecohydrological classes derived from NDVI metrics.
• ecoHydro_attributes_SierraNevada.csv: table with descriptive statistics (mean, SD, min, max) for each of the 64 classes across the three NDVI-based variables.
• README.txt: brief description of the dataset content and file structure.
• metadata_pGDV_dataset.pdf: full metadata including methodological summary, variable descriptions, and legend for map interpretation.

All spatial files use the ETRS89 / UTM zone 30N projection (EPSG: 25830). The data can be visualized in standard GIS software (e.g., QGIS, ArcGIS) and analyzed using geospatial or statistical tools.

## 4 Experimental Design, Materials and Methods

**Fig. 2:**
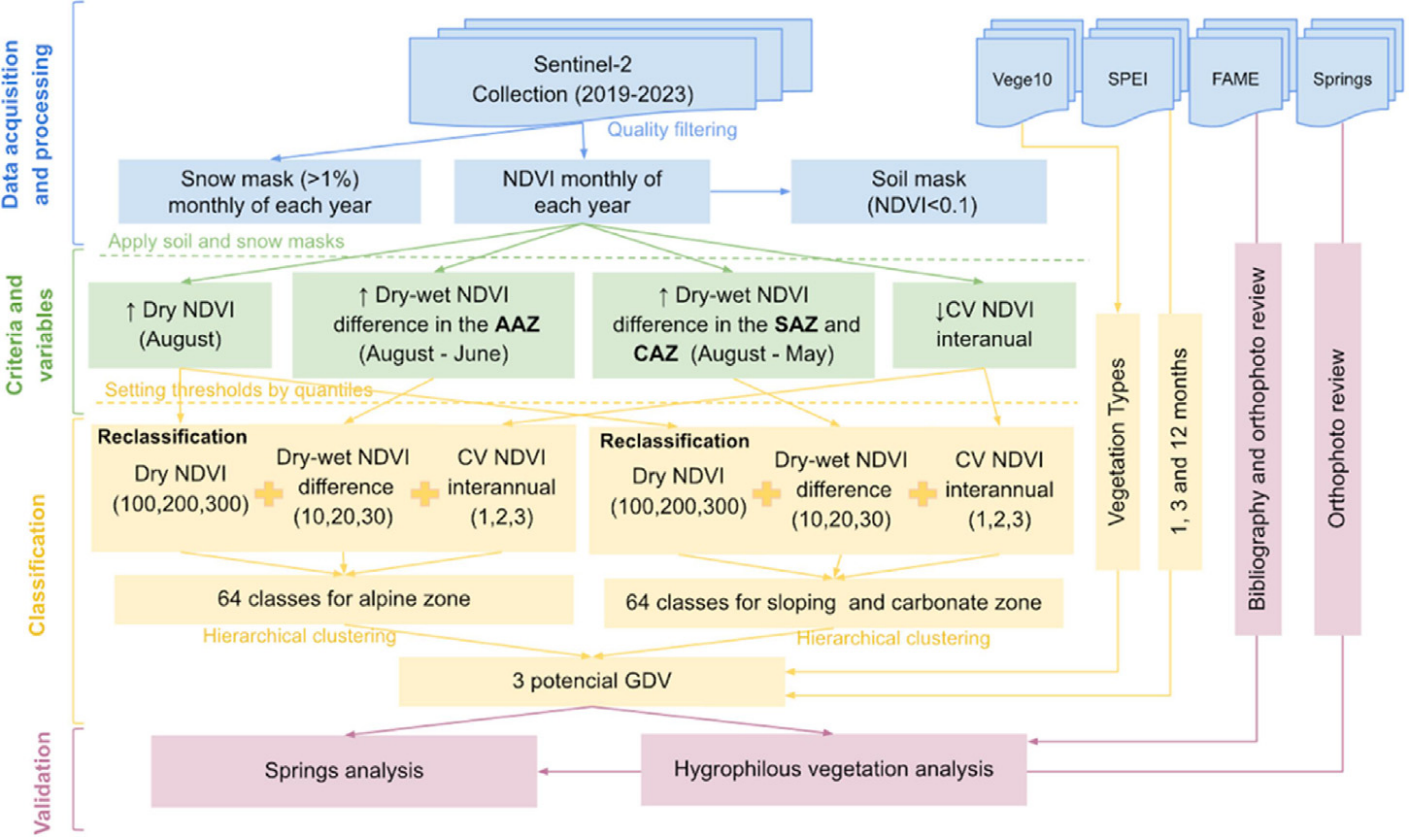
Workflow for generating the pGDV dataset through a multi-step approach integrating remote sensing time series, ecohydrological metrics, and aquifer mapping.

The pGDV dataset was generated through a multi-step approach integrating remote sensing time series, ecohydrological metrics, and existing aquifers maps (Fig. 2). The process was structured in five main phases:

1. Data acquisition: Sentinel-2 Level 2A imagery from January 2019 to December 2023 was obtained from the Copernicus Open Access Hub. A total of 2,537 images covering the Sierra Nevada Protected Area were used. Cloud and snow masking were applied using Sentinel-2 QA bands (MSK_CLDPRB, MSK_SNWPRB), excluding pixels with >10% cloud probability and >1% snow probability. A minimum NDVI threshold of 0.1 was used to mask non-vegetated areas, validated using high-resolution orthophotos (PNOA).
2. Calculation of NDVI-based ecohydrological attributes: Monthly NDVI was calculated using Sentinel-2 bands B4 (red) and B8 (NIR). Three attributes were derived: Dry-season NDVI (NDVI in August), Dry–wet NDVI difference (May/June vs August, altitudinally adjusted), and Interannual NDVI coefficient of variation (CV from 2019–2023)
3. Classification into ecohydrological classes and pGDV levels: Following the functional classification approach proposed by [12], each NDVI-derived ecohydrological attribute was divided into four quartile-based categories, representing gradients of ecosystem functioning. To facilitate class labeling and subsequent interpretation, numerical codes were assigned to each category (e.g., 100–400 for dry-season NDVI, 10–40 for dry–wet NDVI difference, and 1–4 for interannual NDVI CV). The combination of the three attributes resulted in 64 unique ecohydrological classes, each reflecting a distinct functional profile potentially linked to groundwater availability. For each class, summary statistics (mean, SD, min, max) were calculated and used as input for hierarchical clustering (Euclidean distance, complete linkage). The resulting dendrogram was pruned to define three levels of potential groundwater dependence: pGDV1 (low), pGDV2 (moderate), and pGDV3 (high). Separate clustering was applied to low-elevation (SAZ, CAZ) and high-elevation (AAZ) zones.
4. Spatial analysis and mapping: Raster layers were generated at 10 m spatial resolution for both the 64 ecohydrological classes and the three-level pGDV classification. A CSV file was also produced containing descriptive statistics for each class. All layers use the ETRS89 / UTM Zone 30N coordinate system (EPSG: 25830).
5. Tools and software used: NDVI processing and filtering were performed in Google Earth Engine. Statistical and clustering analyses were conducted in R (v4.2.3), using the packages: *raster, sp, terra, rgdal, cluster, factoextra, FactoMineR, NbClust, and ggplot2*. Spatial visualization and map export were done in QGIS Desktop 3.28.4.
6. Partial validation of the pGDV classification: To assess the ability of the pGDV map to detect known groundwater-dependent vegetation, a partial validation was conducted using two spatial indicators: i) Springs georeferenced across seven river catchments (Nechite, Mecina, Bérchules, Alhorí, Bernal, Ohanes), compiled from published sources [13,14] and field data; and ii) Occurrence records of hygrophilous plant species, selected based on expert classifications [15] from the Sierra Nevada Endangered Flora Database (FAME), an official database managed by the Andalusian Environmental Information Network (REDIAM) that compiles validated records of threatened plant species across the region [16].
7. Spatial intersections between these indicators and the pGDV map were used to compute Producer’s Accuracy, focusing on omission errors (i.e., known groundwater-dependent locations not classified as pGDV3). The analysis considered only presence data, as absence records were not reliable for validation purposes. Records with imprecise georeferencing were excluded. A chi-square (χ²) test was used to evaluate whether the spatial association between indicators and pGDV3 class was statistically significant. Analyses were conducted separately by hydrological zone (AAZ, SAZ, CAZ).

## Limitations

Several limitations should be considered when using this dataset. First, the NDVI time series used to derive ecohydrological attributes are based on Sentinel-2 imagery with a 10 m spatial resolution, which may not fully capture narrow riparian corridors or small groundwater discharge areas. Cloud and snow filtering, although applied systematically, may lead to data gaps in high-elevation zones, especially during winter months.

The NDVI threshold of 0.1 used to mask non-vegetated areas may exclude low-biomass ecosystems with some degree of groundwater dependence, such as sparsely vegetated wetlands or early-successional habitats. The classification into pGDV levels is based on remotely sensed proxies, which, although validated using spatial indicators (springs and hygrophilous species), do not directly measure groundwater use.

Vegetation typologies were derived from a reclassification of an existing regional map (VEGE10), which, although detailed, may contain classification uncertainties at ecotones or mixed land cover types. Finally, the validation was based on presence-only data, which limits the ability to assess overall classification accuracy.

Despite these limitations, the dataset provides a consistent and reproducible framework for identifying and comparing groundwater-dependent vegetation in semi-arid mountain environments.

## Ethics Statement

The authors confirm that they have read and follow the ethical requirements for publication in Data in Brief. The current work does not involve human subjects, animal experiments, or any data collected from social media platforms.

## CRediT Author Statement

Javier Cabello: conceptualization, methodology design, formal analysis, investigation, writing - reviwing and editing, and funding. Montserrat Escudero-Clares: methodology, software, data curation, formal analysis, reviewing and editing. Sergio Martos-Rosillo: conceptualization, investigation, writing - reviewing and editing. J. Jesús Casas: conceptualization, investigation, funding, reviewing and editing. Juanma Cintas: software, data curation, review and editing. Thomas Zakaluk: investigation, reviewing and editing. María J. Salinas-Bonillo: conceptualization, formal analysis, investigation, funding, writing and reviewing

## Data Availability

ZENODOMap of potential groundwater-dependent vegetation in the Sierra Nevada Protected Area (Southern Spain) based on NDVI-derived ecohydrological attributes (Original data).
